# Modeling human diseases: an education in interactions and interdisciplinary approaches

**DOI:** 10.1242/dmm.025882

**Published:** 2016-06-01

**Authors:** Leonard Zon

**Affiliations:** 1 Howard Hughes Medical Institute, Stem Cell Program and Division of Pediatric Hematology/Oncology, Boston Children's Hospital, Dana-Farber Cancer Institute, Harvard Medical School, Boston, MA 02115, USA; 2 Department of Stem Cell and Regenerative Biology, Harvard Stem Cell Institute, Cambridge, MA 02138, USA

## Abstract

Traditionally, most investigators in the biomedical arena exploit one model system in the course of their careers. Occasionally, an investigator will switch models. The selection of a suitable model system is a crucial step in research design. Factors to consider include the accuracy of the model as a reflection of the human disease under investigation, the numbers of animals needed and ease of husbandry, its physiology and developmental biology, and the ability to apply genetics and harness the model for drug discovery. In my lab, we have primarily used the zebrafish but combined it with other animal models and provided a framework for others to consider the application of developmental biology for therapeutic discovery. Our interdisciplinary approach has led to many insights into human diseases and to the advancement of candidate drugs to clinical trials. Here, I draw on my experiences to highlight the importance of combining multiple models, establishing infrastructure and genetic tools, forming collaborations, and interfacing with the medical community for successful translation of basic findings to the clinic.

## Abstract (precis)

**Summary:** Animal models have greatly facilitated the process of drug discovery. Len Zon draws on his own experiences to highlight how a cross-model, interdisciplinary approach can bring basic findings to the clinic.

Animal models are fundamental tools in biomedical research. They offer the unique opportunity to investigate the function of genes and pathways and the effects of drugs *in vivo*, bridging the gap between basic science and the treatment of human diseases. Models are invaluable to provide new insight into mechanisms underlying organ function or to establish the pathophysiology of a human disease. A key feature of the models is that they are amenable to manipulation, including via the use of diverse genetic and biochemical approaches and, in some cases, high-throughput drug screens. An animal model must be true to its cause and have the ability to reflect human biology and pathology accurately. This, together with how readily it can be manipulated with the available tools and assays, determines which model or combination of models is most appropriate for exploring specific disease or therapeutic questions.

## Choosing wisely

The choice of animal models in my own lab came down to the need to have a large number of animals for observation, which is key to understanding vertebrate development. Large numbers enable screening of pathways operating during embryogenesis that could play an important role in adult biology. My lab is also interested in understanding the genetics underlying human diseases, including leukemias, cancers and anemias. With these goals in mind, I selected the zebrafish as a primary model system. Hundreds of embryos can be generated from a single set of zebrafish parents, facilitating high-throughput screens. Crucially, the embryos are optically transparent and organs can be viewed under a light microscope. The animals can be mutagenized readily, facilitating rapid screens for genetic mutants with disease phenotypes. My foray into zebrafish research began with the development of a number of blood disease models and, as time went on, we also started using the model for cancer biology, having realized that we could induce tumors in the fish (Jing and Zon, 2011; White et al., 2013). Thus, embryos were plentiful and the genetic tools were already excellent, positioning zebrafish at a sweet spot for understanding disease and exploring therapies.

## You can't play a symphony unless you can play scales

The genome sequence is a valuable asset for any model system. In the early days of zebrafish research, a few individuals stepped up and created crucial reagents for the definition of its genome. We needed libraries of large DNAs (bacterial, P1-derived and yeast artificial chromosomes) and polymorphic markers on the chromosomes (Amemiya et al., 1999). The National Institutes of Health (NIH) developed the Trans-NIH Zebrafish Genome Initiative, which provided the first genetic maps and important tools for analysis and annotation of the zebrafish genome. The zebrafish genome is very repetitive, and it took substantial efforts from the Sanger Center and the community to stitch it together (Howe et al., 2013). It really helped having the large DNA libraries from multiple sources. Sequencing of the zebrafish genome provided an excellent prototype for the dissection of other vertebrate genomes. Having said this, the process was very slow and took years. Once the Sanger Center announced that it would sequence the zebrafish genome, the NIH decided not to participate in sequencing. The lack of competition meant that the process became slow and methodical. I was part of those early discussions at the NIH, and I wish we could have convinced them to fund a parallel effort to the Sanger. Today, genome sequencing is so easy that it is possible to preliminarily characterize the genome of new models. Such characterization allows for immediate analysis of gene function, and will likely significantly reduce the time it takes to optimize a new animal model for a specific research question.

Crucial to the success of a model organism is the development of appropriate infrastructures by dedicated researchers. For instance, animal husbandry is specific to each organism, and so, for zebrafish, the stock centers [such as the Zebrafish International Resource Center (ZIRC; https://zebrafish.org/home/guide.php)] and associated veterinarian staff are important to direct the use of the model. As genomic data are generated, dedicated databases become crucial. We are lucky to have ZFIN as the database for the zebrafish system (http://zfin.org); FlyBase (http://flybase.org) provides a parallel, fantastic resource for investigators working on *Drosophila*. In a world of connectivity, these databases are continually being expanded and optimized. There are new methods to examine conserved genes and pathways, and the processing of data has become easier and easier. Genome browsers, such as those hosted at University California Santa Cruz (https://genome.ucsc.edu/) and the Sanger Institute (http://www.sanger.ac.uk/science/data/zebrafish-genome-project) have become invaluable resources.

## Hard work pays off

Soon after I began working on zebrafish, it became possible to make transgenic fish (Long et al., 1997) and to also do chemical screening in this system (Zon and Peterson, 2005). Transgenics gave us the unparalleled power to visualize organs *in vivo*. We created the first optically transparent adult zebrafish in which we could transplant stem cells or tumor cells and visualize resulting organs (White et al., 2008). Chemical screening, pioneered by Randy Peterson's lab, provided us with a powerful method to look for mutant phenotypes in embryos and even to do suppressor screens to identify drugs that would rescue a mutant phenotype. Recently, the study of adults as well as embryos has become common in the zebrafish system and this furthers the cause for it as an excellent disease model, as illustrated below.

**Fig. 1.**
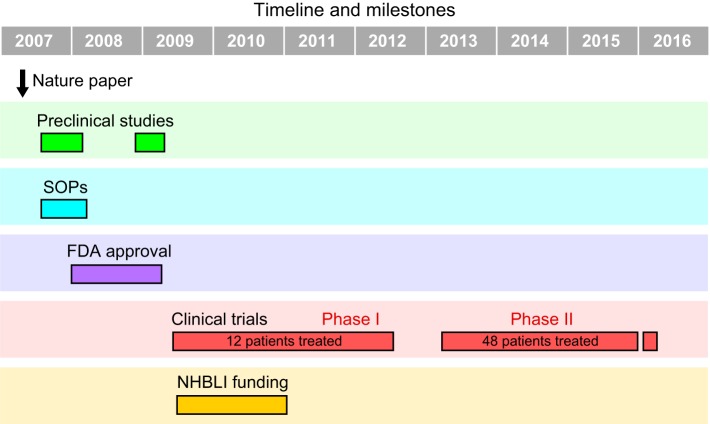
**Timeline of translating a basic discovery in an animal model to the clinic.** Our initial paper on the induction of blood stem cells by PGE2 was published in 2007 (North et al., 2007). We initiated preclinical studies to show that the chemical worked in mouse bone marrow transplants and with human cells in immunodeficient mice. We developed standard operating procedures (SOPs). We received FDA approval to initiate the Phase I clinical trial. 12 patients were treated. We received NIH funding for the trial [National Heart, Lung, and Blood Institute (NHBLI)]. A company, FATE Therapeutics, participated in the trial. A Phase II trial was done with 48 patients.

**Fig. 2.**
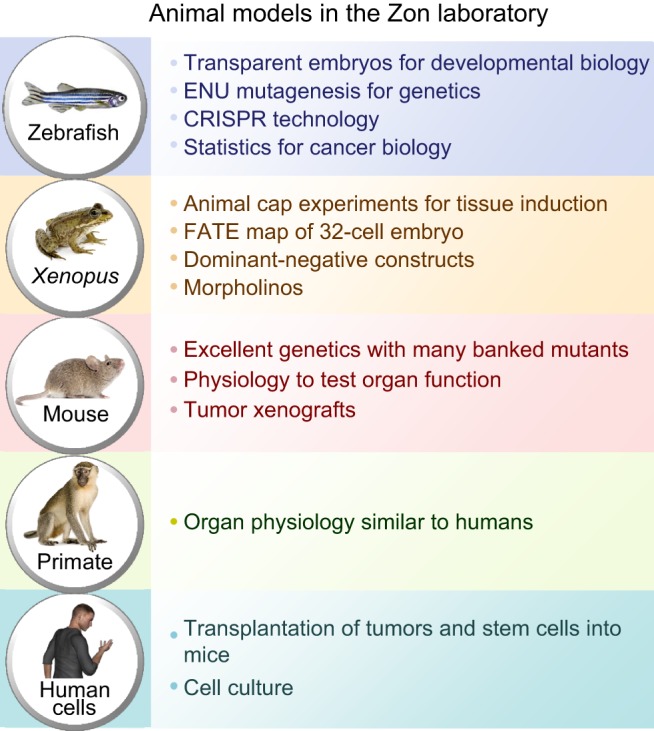
**Models that have been used in the Zon lab.** Key attributes of different animal models used for the study of human diseases are highlighted.

Our early zebrafish work included the discovery of five previously undescribed human blood diseases, based on the analyses of mutant fish found by ENU mutagenesis screens (North and Zon, 2003). The human orthologs of zebrafish genes mutated in our anemic mutants were found to be the causative genes in several newly described human diseases. We also uncovered many findings that defined the process of blood development and gave insight into cancer formation. Using chemical screening, we were able to pinpoint two drugs that made it from the initial assay in embryos to successful testing in adults and, ultimately through to a human clinical trial. In the first case, we found that prostaglandin E2 (PGE2) can increase the number of stem cells in the developing aorta and also expand hematopoietic cells upon transplantation in adult zebrafish (North et al., 2007). We had thus discovered the first small molecule to increase a stem cell pool, with implications for the treatment of individuals with bone marrow failure and following bone marrow transplantation. From publication of the initial findings through to Phase II clinical trial – still ongoing – the route to translation has taken almost 10 years (Fig. 1). This is slightly faster than traditional drug development by pharma but still leaves an opportunity to accelerate the process. In another study, we examined the ability to block neural crest development as a potential therapy for melanoma (White et al., 2011; Phillips and Westerfield, 2014). This led to the discovery that leflunomide, a drug commonly used to treat arthritis, could eliminate neural crest development based on transcriptional pausing. Additional studies in other animal models were required in both of these cases to be able to bring the basic findings to the clinic (Fig. 2).

## Model envy and combinatorial approaches

The mouse is a wonderful system that is complementary to the zebrafish and has over 100 years of science associated with it. The mouse allows one to establish a principle in a mammalian species, strengthening the relevance to humans. Building on our PGE2 story, we were able to show that this compound can enhance the engraftment of blood stem cells in mouse bone marrow transplants (North et al., 2007). This work made use of established assays and cell surface markers that facilitate flow cytometry in the mouse. It would be nice for the fish to someday approach the mouse in terms of the availability of such defined assays.

I actually started my lab with a primary focus on the *Xenopus laevis* system and cloned a homoebox gene that stimulates hematopoiesis (Mead et al., 1996). I was drawn to the established developmental biology of this model; nothing beats looking at tissue induction with the animal cap assay. At the time, there were no morpholinos and no genetic tools, and so I stopped working on *Xenopus*. Nowadays, powerful genetic and transgenic tools are available for the community working on *X. topicalis*. Its unique developmental biology and the availability of these tools now makes it a very useful model.

Other models have tremendous value for particular experiments. The genetic tools available to study worms and flies are amazing, and clonal analysis – the study of the progeny of single cells – is unparalleled in these models. If you are interested in a signaling pathway, the ability to trace individual clones is incredible. Experiments involving the rescue of mutant phenotypes with the relevant human gene have demonstrated the remarkable conservation of biological functions and pathways across vertebrates. Given my interest in hematopoiesis, I'm intrigued by protochordates such as *Ciona* that have circulating blood cells during larval life, but lose them in adulthood (Kourakis and Smith, 2015). Studying blood development is thus possible in protochordates, and the low numbers of orthologs compared to mainstream vertebrate models is an attractive feature of these organisms. Physiology is probably best studied in mammals, such as rats, pigs or primates. Often, primates are used for the final drug testing stage before moving a therapy to humans. A number of aging models have emerged recently, including the rapidly aging killifish (Kim et al., 2016), further illustrating that a wide range of animal models is important to address diverse research questions.

I'd fondly describe the first zebrafish group, set up in Oregon in the early 1980s, as a bunch of hippies – there was a clear message of working together – and they taught me the value of the community spirit in this field. I'm finding that all of the animal model communities are now coming together in a big kumbaya, illustrated by the 2016 Allied Genetic Society of America (GSA) meeting, which brings together seven different meetings for the first time. It is increasingly recognized that we need to exploit the best attributes of each model system to understand how processes and pathways operate (Fig. 2). Understanding the cross-section of these models at the molecular and genome level, studying tissue form and function, will be the future.

## Time to translate

The translation of new therapies ultimately requires the evaluation of human cells. We used human tumor xenographs to show that leflunomide has antitumor activity, providing strong preclinical data to support its application in the treatment of cancer. Because leflunomide was already approved by the US Food and Drug Administration (FDA) (for the treatment of arthritis), we could immediately go forward to a Phase II clinical trial. Combining different models made the study extremely powerful. In my lab, we were lucky enough to have zebrafish, mouse, and human cell-based models available to us. Similarly, translation of PGE2 involved the demonstration of its activity on human CD34 cells from peripheral blood or from cord blood, and enhanced engraftment activity of PGE2-treated stem cells in immunodeficient mice (Goessling et al., 2011). With this strong preclinical data, we were able to go to the FDA for approval of an Investigational New Drug (IND) application for PGE2 and the treatment of leukemia through cord blood transplantation.

Being able to generate preclinical data with human cells and two animal models made investigators realize that the zebrafish was a great entryway to discovery and that the mouse was a wonderful way of proving that a point was relevant to mammalian species. The clinical world was alerted to these findings at medical conferences, and key individuals who are involved in clinical trials were supportive of this work. This ultimately led to its translation: the leflunomide trial is now underway. For PGE2, a Phase I clinical trial demonstrated that, in 10 out of 12 patients treated with a cord blood transplant in which two cord bloods were used, the treated cord was the one that preferentially engrafted (Cutler et al., 2013) (Fig. 1). A Phase II clinical trial was recently reported at the American Society of Hematology with similar observations. In addition, there has appeared to be less graft-versus-host and also less viral reactivation, perhaps indicating that the T-cells have also been stimulated and might produce an anti-viral response. The trials indicate that PGE2 has activity in human hematopoiesis, and additional trials are now being planned to define the path towards FDA approval.

In summary, the analysis of multiple animal models provides an effective mechanistic framework for translational research. To start a clinical trial, it is important to impress upon colleagues that your basic ideas work in multiple systems. Importantly, we were able to demonstrate that the mechanism of action of PGE2 in embryos is rather similar to that in the adult, which convinced us – and others – of the potential to use embryos as a method to define pathways and identify new therapeutic routes. We have also been able to show that cancer-related phenotypes, such as the activation of the neural crest in melanoma, in adults replicate what happens in embryogenesis. Clinical investigators are very impressed by the ability to probe both embryos and adult zebrafish, and this attribute means that more investigators will inevitably come into the zebrafish field to examine the basic biology of diseases and identify new therapeutics.

## The world is your oyster (or zebrafish)

The future of zebrafish research is very bright, and I'm excited to see how this model will participate in the next decade of biomedicine. We have come very far since the hippie days in Oregon in the late 1980s. As a premier genetic system, amenable to CRISPR and other gene-editing strategies, the creation of new zebrafish models will continue to happen at light-speed. Investigators interested in regenerative medicine are looking to the zebrafish to understand the regeneration of the immune system and many other tissues. The biological knowledge we now have for each zebrafish organ is very rich. Neural circuitry is being evaluated directly, and optogenetics has been applied. The intersection of human genetics and the tractability of the zebrafish model allow investigators to rapidly establish gene function. The Environmental Protection Agency (EPA) frequently uses zebrafish to assay potential toxicity of chemicals. An argument could be made that zebrafish is the best system for chemical screens, although we are just at the beginning of translating drugs found to be effective in fish to humans.

To continue this trend, the field must invest in reagents that make it equivalent to the mouse system. For instance, we recently created the only monoclonal antibody targeted to an immune cell of the zebrafish (unpublished results, Zon laboratory). The mouse and human systems have hundreds of antibodies that mark the respective immune systems. Although tedious, we need to strive to make zebrafish every bit as capable as the mouse system.

## A big group hug

There are a number of groups who are dedicated to the study of zebrafish. There is the International Zebrafish Society (http://izfs.org/) and the European Society for Zebrafish Models in Biology and Medicine (https://www.eufishbiomed.kit.edu/). These groups run annual meetings. The Zebrafish Disease Model society (http://zdmsociety.org/) was recently started with the specific purpose of bringing together scientists, clinical researchers and doctors interested in using this model to understand disease and discover therapeutics. It is helpful to have community-driven initiatives for the ultimate success of a model.

I am proud to be a physician scientist (Zon and Cagan, 2014). I have always taken care of patients while running a laboratory. My clinical practice is winding down, but I enjoy being a Hematologist-Oncologist. I have had to deliver tough news to patients, and I occasionally say “someday there will be a specific pill for this cancer”. Drug discovery takes a long time; I'd estimate 10 years from discovery to a Phase III clinical trial. Animal studies could accelerate this process. We need to develop a blueprint for developing new therapies. Attempts have been made using *Drosophila* as a discovery system for anti-cancer therapies (Das and Cagan, 2013). We can learn from this example how to develop a precision-medicine approach, and extend this to vertebrate systems. A ‘mouse hospital’ already exists in the Pandolfi laboratory to model how patients are taken care of. Cross-model interactions offer the best chance to move rapidly in translational medicine. Can we satisfy an NIH consortium dream – moving from flies and fish to mouse quickly, and then to human cells? Fulfilment of this dream will require coordination, and the Allied GSA meeting could be the beginning of increasingly fruitful collaborations that set the wheels in motion.
